# Interdependence of balance mechanisms during bipedal locomotion

**DOI:** 10.1371/journal.pone.0225902

**Published:** 2019-12-04

**Authors:** Tyler Fettrow, Hendrik Reimann, David Grenet, Elizabeth Thompson, Jeremy Crenshaw, Jill Higginson, John Jeka

**Affiliations:** 1 Kinesiology and Applied Physiology, University of Delaware, Newark, DE, United States of America; 2 Department of Physical Therapy, University of Delaware, Newark, DE, United States of America; 3 Department of Kinesiology, Temple University, Philadelphia, PA, United States of America; 4 Department of Physical Therapy, Temple University, Philadelphia, PA, United States of America; 5 Department of Mechanical Engineering, University of Delaware, Newark, DE, United States of America; University of Utah, UNITED STATES

## Abstract

Our main interest is to identify how humans maintain upright while walking. Balance during standing and walking is different, primarily due to a gait cycle which the nervous system must contend with a variety of body configurations and frequent perturbations (i.e., heel-strike). We have identified three mechanisms that healthy young adults use to respond to a visually perceived fall to the side. The lateral ankle mechanism and the foot placement mechanism are used to shift the center of pressure in the direction of the perceived fall, and the center of mass away from the perceived fall. The push-off mechanism, a systematic change in ankle plantarflexion angle in the trailing leg, results in fine adjustments to medial-lateral balance near the end of double stance. The focus here is to understand how the three basic balance mechanisms are coordinated to produce an overall balance response. The results indicate that lateral ankle and foot placement mechanisms are inversely related. Larger lateral ankle responses lead to smaller foot placement changes. Correlations involving the push-off mechanism, while significant, were weak. However, the consistency of the correlations across stimulus conditions suggest the push-off mechanism has the role of small adjustments to medial-lateral movement near the end of the balance response. This verifies that a fundamental feature of human bipedal gait is a highly flexible balance system that recruits and coordinates multiple mechanisms to maintain upright balance during walking to accommodate extreme changes in body configuration and frequent perturbations.

## Introduction

Investigations of human balance control have focused primarily on standing, either during unperturbed quiet stance [1, 2] or in response to mechanical [3] and sensory perturbations [4, 5]. Surprisingly, the literature on balance control during walking is relatively sparse, despite the fact that most falls occur during walking [6–8]. One explanation for this disparity is that walking entails many behaviors that are not relevant to standing, fostering investigations into navigation [9, 10], obstacle avoidance [11] and metabolic efficiency [12]. However, we argue that balance control during walking requires an analysis that differs markedly from standing balance, for a number of reasons. First, the temporal window over which the human body propels itself (i.e., the gait cycle) allows for distinct balance responses to be initiated sequentially. Second, body configuration changes dramatically over the gait cycle (e.g., single stance to double stance), resulting in balance responses that are highly constrained by this reconfiguration. Third, the nervous system must respond not only to external disturbances while walking but respond to disturbances that are inherent to the gait cycle itself (e.g., heel-strike). In this regard, walking is often conceived as a hybrid dynamical system, combining elements of both continuous and discrete dynamics, requiring a different form of control than standing. Here we determine how the human nervous system coordinates multiple balance mechanisms during walking to determine whether such mechanisms are interdependent as they unfold over the gait cycle.

We have identified three basic mechanisms following visual perturbations in the medial-lateral direction that shift the CoP in the desired direction, the lateral ankle mechanism, the foot placement mechanism, and the push-off mechanism [13]. We provide a description of these basic control mechanisms available to healthy adults, and determine whether the mechanisms are temporally coordinated throughout the gait cycle. We expect all three mechanisms to be interdependent to produce an overall balance response.

The lateral ankle mechanism refers to the generation of ankle inversion/eversion torque during the single stance phase. Rolling the ankle while the foot is on the ground can shift the CoP under the foot. The goal is to shift CoP in the direction of the perceived fall under the stance foot. The lateral ankle mechanism is able to act on the CoP at virtually any time during the gait cycle, and is the main means of modulating the CoP under the stance foot during sustained locomotion. However, the magnitude of CoP modulation that the lateral ankle mechanism can produce is constrained to the width of the foot or shoe.

The foot placement mechanism is another mechanism that can shift the CoP. The swing foot is shifted in the direction of the perceived fall, and on heel-strike, shifts the CoP. Foot placement is the most commonly reported measure of dynamic stability [14, 15]. Much of the balance and walking literature focuses on swing foot placement in terms of overall step width or step width variability [16–18]. The foot placement mechanism is different from step width, which assumes an increase in step width. Here we are interested in the control of foot placement in order to modulate the CoP in a particular direction, which can result in an increased or a decreased step width.

The push-off mechanism refers to the modulation of ankle plantarflexion angle during double stance. Very few studies have recognized the push-off mechanism’s role in balance. Ankle plantarflexion torque has been shown to change as a result of bipolar, binaural galvanic vestibular stimulation [19] and anterior-posterior mechanical perturbations [20]. Only recently has the push-off been verified to have a functional role in balance control in the medial-lateral direction [21, 22]. In response to a visually perceived fall to the side, we observed a direction-dependent modulation of the stance leg ankle plantar/dorsiflexion angle [13].

The three identified balance mechanisms are the main available methods to make fine motor adjustments to the CoP and CoM in order to maintain balance while walking. Given that multiple mechanisms are available to the CNS to maintain balance, we analyzed whether these mechanisms are functionally related. For example, modeling results suggest that without any CoP modulation during the stance phase following a sensory perturbation, a 60% larger foot placement is needed to maintain balance in the medial-lateral direction [23]. Here we determine if the human nervous system coordinates the balance response throughout the gait cycle by testing whether these mechanisms are interdependent to produce an overall balance response.

## Methods

Portions of this data and detailed data collection methods have already been published [13], where we focused entirely on group averages. Here we focus on the balance mechanisms on every step. Twenty healthy young subjects (11 female, 22.8 ± 4.1years, 75.2 ± 17.9kg) volunteered for the study. Subjects provided informed written consent to participate. Subjects did not have a history of neurological disorders or surgical procedures involving legs, spine or head. The experiment was approved by the Temple University Institutional Review Board.

### Experimental design

After explaining the experiment, obtaining consent and placing markers and EMG sensors, subjects first walked for 15 min on the self-paced treadmill in the virtual environment to adapt to this experimental setup. We then stopped the treadmill and told the subjects that we would now perturb their sense of balance by modifying the virtual scene, and asked them to cope with this perturbation “normally” and keep walking forward. Data collection blocks consisted of two alternating phases for metronome and stimulus. During metronome phases, lasting 30 s, subjects were provided an auditory metronome at 90 bpm and asked to use this as an “approximate guideline” for their footsteps, both during metronome and stimulus phases. During stimulus phases, lasting 120 s, the metronome was turned off, and subjects received visual fall stimuli as described above. Data were collected during stimulus phases. Each subject performed four blocks of walking, each block consisting of five metronome and five perturbed phases, always starting with metronome phases, for a total of 12.5 min per block. After each block, the treadmill was turned off and subjects were offered a break. This protocol was implemented in a custom Labview program that sent the head position, treadmill speed and rotation angle to the computer controlling the virtual environment implemented in Unity3d (Unity Technologies, San Francisco, CA, USA) via User Datagram Protocol communication. The visual rotation angle and treadmill speed were saved in Labview at 100 Hz.

Subjects walked on a split-belt, instrumented treadmill within a virtual environment projected onto a dome surrounding the treadmill by ∼180 degrees (Bertec Inc., Columbus, Ohio, USA). The treadmill was self-paced, using a nonlinear PD-controller in Labview (National instruments Inc., Austin, TX, USA) to keep the middle of the posterior superior iliac spine markers on the mid-line of the treadmill. Perturbations consisted of a rotating virtual environment scene with an acceleration of 60° s^−2^ for 600 ms around the anterior-posterior axis of the midline of the floor, inducing a feeling of falling to the side. The perturbations were provided randomly on a right or left heel-strike (stimulus trigger), and rotation direction were randomized rotate clockwise or counter-clockwise. Perturbations in which the environment rotates clockwise corresponds to the visual sensation of a fall in the opposite direction, counter-clockwise or to the left. Similarly, a counter-clockwise rotation of the environment corresponds to the sensation of a clockwise fall, or to the right. We will refer to these as fall stimulus to the right and fall stimulus to the left. Furthermore, we will assume body symmetry and pool data for cases where the direction of the perceived fall is towards the triggering leg, i.e. right heel-strike triggering fall stimulus to the right and left heel-strike triggering fall to the left, and cases where the direction of the perceived fall is away from the triggering leg, i.e. right heel-strike triggering fall stimulus to the left and left heel-strike triggering fall stimulus to the right. The resulting rotation of 10.8° was then held constant for 2,000 ms, before being reset to neutral rotation with uniform speed over 1,000 ms. After resetting to neutral rotation, a randomized interval of 10-13 steps elapsed before the next stimulus was triggered. Heel strikes were identified as downward threshold crossings of the vertical heel-marker position. The threshold was set to the vertical heel-marker position of each foot during quiet standing, plus 3 mm.

Reflective markers were placed bilaterally on the feet, lower legs, thighs, pelvis, torso, head, upper arms, forearms, and hands of the subject, using the Plug-in Gait marker set [24] with six additional markers on the anterior thigh, anterior tibia, and 5th metatarsal of each foot for a total of 45 markers. Marker positions were recorded at 250Hz using a Vicon motion capture system with nine cameras. Ground reaction forces and moments were collected at 1,000 Hz from both sides of the instrumented split-belt treadmill. Forces and moments were transformed into a common coordinate frame and then used to calculate the whole-body CoP [25].

### Data management and organization

Kinematic data were low pass filtered with a 4th order Butterworth filter at a cut-off frequency of 10Hz. Small gaps in the marker data of up to 100ms length from occlusions were filled using cubic splines. Time points with remaining marker occlusions were excluded from further analysis. From the marker data, we calculated joint angle data based on a geometric model with 15 segments (pelvis, torso, head, thighs, lower legs, feet, upper arms, forearms, hands) and 38 degrees of freedom (DoF). We estimated the hip joint centers based pelvis landmarks [26, 27], and the knee joint centers and knee flexion rotational axes from reference movements using the symmetrical axis of rotation approach [28]. We performed inverse kinematics by minimizing the distance between the measured and the model-determined marker positions [29]. This optimization was performed first for the six pelvis DoFs, which formed the root of the kinematic tree, then for the six DoFs at the lumbar and cervical joints, and last for each of the arms and legs separately. We estimated the body center of mass (CoM) positions based on estimated segment CoM locations [30] and the inverse kinematics and calculated CoM velocities and accelerations using numerical derivation by time.

We identified heel strike events for each foot by finding negative peaks in the vertical positions of the heel markers with minimal inter-peak distances of 250 ms and peak prominence greater than 2 cm, and push-off events as the first peak in the vertical velocity of the 2nd metatarsal marker with a prominence greater than 0.35 ms^−1^ after each heel strike. We visually inspected the result of this automatic identification and applied manual corrections in the rare cases where events were misidentified.

All data between heel-strikes were normalized to 100 time steps. We subtract the *control* mean from all data, including *control*, for every subject. The control steps are defined as the two steps preceding the heel-strike that triggers the visual perturbation. All data between heel-strikes were normalized to 100 time steps. All of the data here are represented as a change in response from the average of the *control* steps.

The experimental design had four distinct stimulus conditions, where each heel strike could trigger a stimulus to the left or right. Here we assume anatomical symmetry and group the conditions that are anatomically similar (i.e. [Right heel strike triggers Stimulus Right, Left heel strike triggers Stimulus Left]). Previous analysis of the data set indicate asymmetries between feet are not present [13]. We also analyzed how the mechanisms are coordinated during *control* steps. This leaves three conditions to analyze, stimulus *towards* trigger foot, stimulus *away* from trigger foot, and *control*. After processing, we were left with 1947 steps for *Stimulus Towards*, 1930 steps for *Stimulus Away*, and 3854 steps for *Control*.

### Quantifying balance mechanisms

Comparing the use of the balance mechanisms on every step requires that the mechanisms are summarized by a single value. We use the following definitions to quantify the use of each balance mechanism on every step:

#### Lateral ankle mechanism

The lateral ankle mechanism is defined as the integrated response from *control* steps of the medial-lateral distance between CoP and CoM during single stance. The integration is performed during single stance to avoid the contribution of the foot placement mechanism on the CoP. Throughout the text we refer to this variable as Ankle.

∫ΔCoP-CoM (1)

#### Foot placement mechanism

We define the foot placement mechanism by the medial-lateral response of the swing leg heel position at heel strike, relative to the initial medial-lateral position of the trigger foot heel strike. Throughout the text we refer to this variable as Foot.

Δ(TriggerFootHeelPosition-SwingFootHeelPosition) (2)

#### Push-off mechanism

We define the push-off mechanism as the integrated response from *control* steps of the trigger foot ankle dorsiflexion/plantarflexion angle during the second double stance phase following stimulus trigger. Throughout the text we refer to this variable as Push.

∫Δplantarflexion (3)

### Statistical analysis

We confirmed the assumptions of normality and homoscedasticity by visual inspection of the residual plots for each combination of variables. Linear mixed models were used for each pair of mechanisms to assess interdependence. For each pair of mechanisms, we fitted a linear mixed model and performed an ANOVA to analyze the interdependence of the mechanisms and interaction of stimulus direction, using Satterthwaite’s method [31] implemented in the R-package lmerTest [32]. We used R (R Core Team, 2013) and lme4 [33] to assess the significance of slopes of the regressions for each pair of mechanisms. Confidence intervals were calculated for the slopes for each stimulus direction using the function confint. The outcome and predictor variables for each model were chosen based on temporal order (i.e. Ankle is used prior to Foot, and Foot prior to Push). We included stimulus direction as a predictor and allowed random intercepts by Subject and random slopes. The following model is an example for assessment of each pair of mechanisms:

Foot∼Ankle+direction+Ankle*direction+(1+Ankle|Subject) (4)

For visual purposes, we perform a least-squares fit for all pairs of mechanisms. Pearson Correlation R^2^ values were calculated for each balance mechanism pair to provide an indication of the strength of the relationship.

## Results

All figures show data averaged from eighteen out of twenty subjects. We removed two subjects from this analysis. These two subjects expressed R^2^ values two standard deviations lower than the mean for Ankle-Foot interdependence. Including the subjects resulted in non-converging of the mixed-models in R.

**Fig 1:**
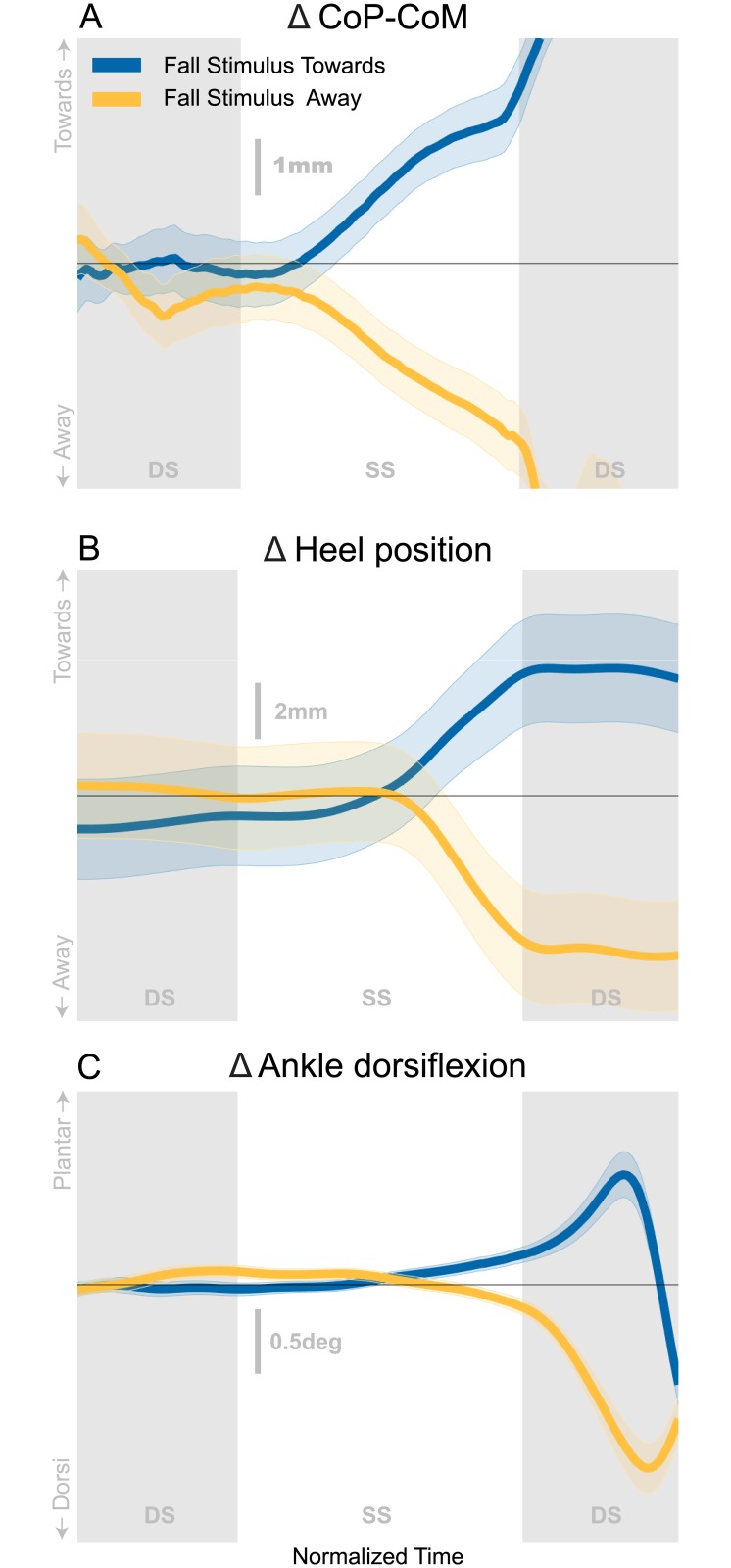
Balance mechanisms in response to a perceived fall to the side. The average difference from control (Δ or response) for three major balance mechanisms are displayed for Stimulus Towards (Blue) and Stimulus Away (Yellow) encased by the 95% confidence interval. Double stance (DS) is displayed as the grey shaded areas. Normalized time represents a total of 832 ms with single stance time indicating 388 ms. The visual scene rotation begins on the onset of the first double stance and continues for 600 ms. Towards and away refer to direction with respect to the triggering leg.

The balance response to a perceived fall laterally (towards and away from trigger foot) consists of a combined response of the lateral ankle mechanism, the foot placement mechanism and the push-off mechanism, as shown in Fig 1. All mechanisms are shown as time series to illustrate their temporal order. After onset of the stimulus on the trigger foot heel strike, the lateral ankle mechanism is the first to act (Fig 1A), on average approximately 350 ms (∼33% of single stance phase) post-stimulus where the *Stimulus Towards* and *Stimulus Away* deviate away from each other. The activation of the lateral ankle musculature leads to a CoP shift towards the direction of the perceived fall, for both *Stimulus Towards* and *Stimulus Away*. Fig 1B shows the initiation of the foot placement mechanism begins around 450 ms (∼60% of single stance phase), represented by the change in swing foot heel position deviating from the control steps towards the end of single stance. This shift starts prior to heel strike, but does not substantially affect the CoP until heel strike. The foot is placed in the direction of the perceived fall. The push-off modulation begins around mid-stance (∼450-550 ms or ∼60-85% of single stance phase), but the majority of the angle change occurs during the second double stance following the stimulus onset (Fig 1C). We observed a plantarflexion response for *Stimulus Towards* and a dorsiflexion response for *Stimulus Away*.

**Fig 2:**
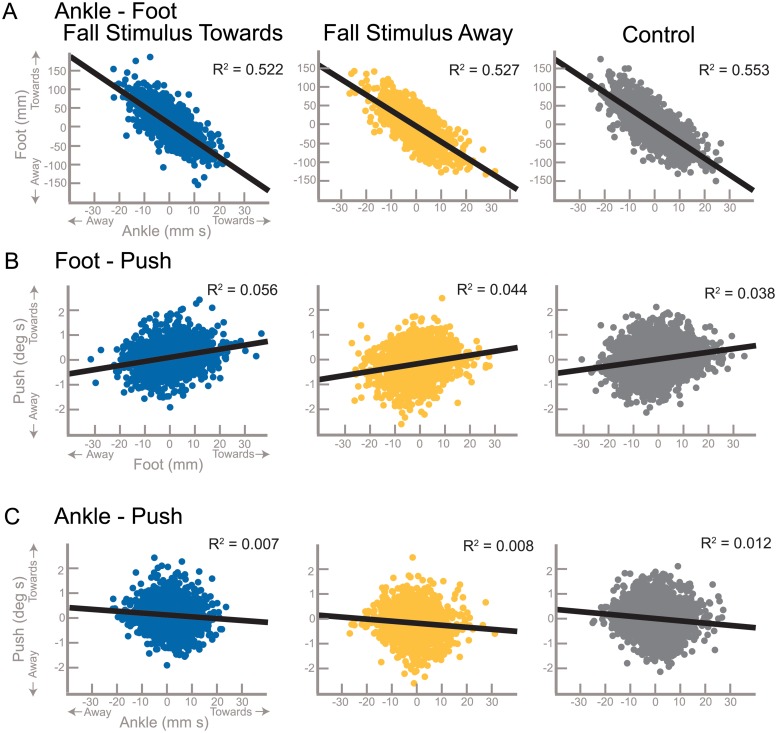
Correlations between the balance mechanisms. The relationship between the three major balance mechanisms are displayed for each stimulus condition with least-squares fit and corresponding Pearson correlation R^2^ values. Each data point represents the combination of the balance mechanisms displayed on the axes for that figure. Towards and away refer to direction with respect to the triggering leg.

Fig 2 illustrates the interdependence between pairs of mechanisms by displaying the use of each mechanism for a given gait cycle as individual data points (See Methods: Quantifying Balance Mechanisms). The correlation between the foot placement and the lateral ankle mechanism is the strongest relative to the other mechanism pairs (Fig 2A) for *Stimulus Towards*, *Stimulus Away* and *Control*, demonstrating a negative interdependent relationship for all conditions (see Tables 1 and 2). A larger response of the lateral ankle results in a smaller foot placement response (and vice versa). A positive but weak correlation exists between the push-off and foot placement mechanisms, for *Stimulus Towards*, *Stimulus Away*, and *Control*, Fig 2B), suggesting that Foot Placement and Push-off are also interdependent (see Tables 1 and 2). Smaller foot placement leads to smaller push-off (and vice versa). Finally, the push-off and lateral ankle mechanisms demonstrate a negative trend *Stimulus Away* (see Fig 2C and Table 2). *Stimulus Towards* and *Control* did not have slopes significantly different from zero, yet trended towards a negative relationship, suggesting the lateral ankle and push-off mechanisms are very weakly interdependent.

**Table 1:** Results of the mixed model output indicating which factors have a significant effect on the outcome variable or dependent variable (see statistical analysis section).

	Fixed-Effect	fNumerator Df	Denominator Df	F	p
Foot	Ankle	1	18	792.47	**<0.0001**
	Direction	2	7710	178.57	**<0.0001**
	Ankle*Direction	2	7684	2.98	0.051
Push	Ankle	1	17	7.59	**0.0133**
	Direction	2	7711	138.27	**<0.0001**
	Ankle*Direction	2	7714	0.36	0.697
Push	Foot	1	17	34.15	**<0.0001**
	Direction	2	7708	106.27	**<0.0001**
	Foot*Direction	2	7712	1.77	0.171

**Table 2:** Results of the mixed model output indicating which factors have a significant effect on the outcome variable or dependent variable (see statistical analysis section).

	Stimulus Towards		Stimulus Away		Control	
Regression Tested	lower	upper	lower	upper	lower	upper
Ankle(mm s)-Foot(mm)	**-6.94**	**-5.34**	**-6.2**	**-5.27**	**-6.762**	**-5.261**
Foot(mm)-Push(deg s)	**0.0018**	**0.007**	**0.003**	**0.006**	**0.001**	**0.006**
Ankle(mm s)-Push(deg s)	-0.029	0.006	**-0.024**	**-0.003**	-0.031	0.002

### Interdependence

**Fig 3:**
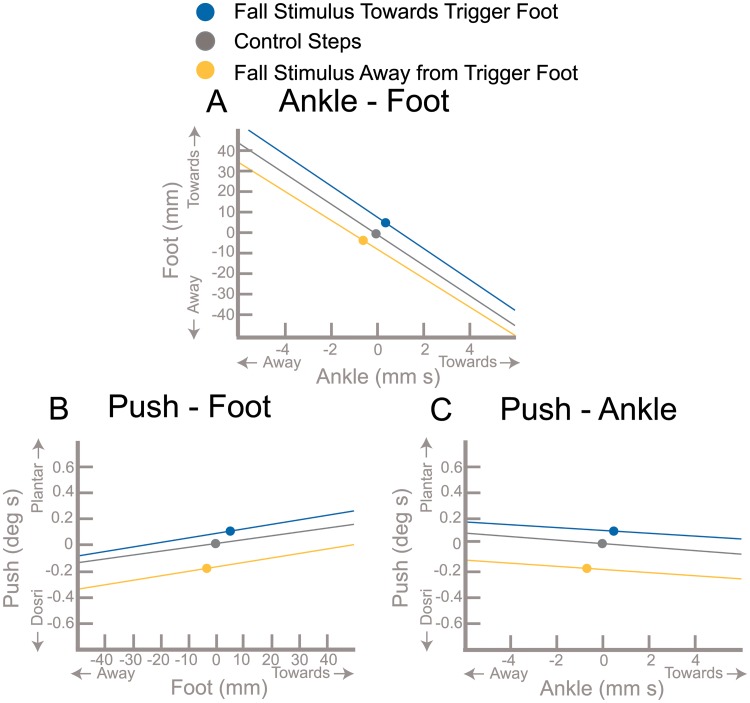
Average correlations between the balance mechanisms. The average for each combination of balance mechanisms for each condition are displayed as solid dots. The least-squares fits are also displayed. Towards and away refer to direction with respect to the triggering leg.

The point clouds of the balance mechanisms across the three stimulus directions in Fig 2 are difficult to distinguish visually. When we remove individual data points from Fig 2 and only include the means and least-squares fits in Fig 3, a clear separation of the stimulus conditions is evident. Balance responses are toward the trigger foot, relative to control steps, when the stimulus is towards the trigger foot. Likewise, in the *Stimulus Away* conditions, balance responses are further away from the trigger foot compared to control steps. This effect of stimulus direction is consistent with results previously published by [13] and confirmed by significance of fixed-effect term Direction in first three mixed-models (Table 1). The fixed-effect term Ankle*Direction tests the change in slope of the models by stimulus direction. No mechanism pair had significantly different slopes across condition, including Ankle-Foot, Ankle-Push, and Foot-Push (see Table 1).

## Discussion

There is a high degree of variability in human gait, even in the control of balance, but we show that the nervous system is able to overcome this variability by making adjustments throughout the gait cycle through the coordination of balance mechanisms. Considering the large number of degrees of freedom involved in bipedal locomotion and resultant body configuration that varies with each step, flexible implementation of balance mechanisms may be fundamental for stable, economical gait.

We have identified three basic balance mechanisms that are used to respond to a perceived fall to the side during locomotion as well as during unperturbed walking. The average balance response as shown in Fig 1 suggests the use of all mechanisms regardless of stimulus direction. The lateral ankle mechanism modulates the center of pressure (CoP) under the stance foot. Change in foot placement then moves the CoP after a modulation of foot position during the swing phase of the gait cycle. We also observed a change in ankle plantarflexion angle depending on the direction of the stimulus within the first gait cycle, indicating that push-off modulation is another mechanism used to respond to a perceived fall to the side. Our conclusions are: 1) balance mechanisms during walking are interdependent; 2) the push-off mechanism is used to make subtle adjustments to balance, but is not strongly interdependent with the other mechanisms; and 3) the use of each balance mechanism alone may be inaccurate (as evidence by common overshooting and undershooting of the lateral ankle mechanism), but the nervous system is able to adjust by using a variety of balance mechanisms throughout the gait cycle.

### Interdependence

The combination of the mechanisms throughout the gait cycle have a compounding effect on balance. In non-perturbed gait, balance throughout the gait cycle may be conceived as a zero-sum scenario. Fig 1 shows that, on average, there are three balance mechanisms used to respond to a perceived fall to the side while walking. The focus of this study was to unpack how all three mechanisms are interdependent to create an overall balance response throughout the gait cycle.

All pairs of balance mechanisms are significantly correlated with one another, despite small R^2^ values, according to the linear mixed model results. Two relationships were negatively correlated: lateral ankle—foot placement and push-off—foot placement. With these two negative relationships, it follows that foot placement and push-off are positively related. Given the disparity in the strength of the correlations between balance response pairs, it seems that the initial response to a disturbance is critical to the overall balance response, leaving the push-off to play a small but systematic modulating role that does not depend strongly on the responses previous to its initiation. Although an interaction between the lateral ankle and foot placement mechanism has been observed previously [34, 35], these reports suggest the lateral ankle mechanism adjusts inaccuracies in foot placement. The current results suggest that foot placement also adjusts to inaccuracies in the lateral ankle mechanism, highlighting an interdependent relationship. The lateral ankle mechanism commonly overshoots or undershoots the required modulation, as evidence by the range of values observed in Fig 2A. This could be viewed as inaccurate use of the lateral ankle mechanism, or be a result of the natural variability in gait and adjustment to the preceding inaccurate foot placement. The subsequent mechanisms (foot placement and push-off) are able to adjust for this inaccuracy or variability.

The current results indicate that there is a third mechanism involved in the coordination between balance mechanisms. The push-off is typically thought to contribute to propulsion, but we believe the push-off is also used as a subtle adjustment to medial-lateral balance, for two reasons. 1) We observe a systematic modulation of ankle plantarflexion angle following a medial-lateral (ML) balance perturbation. It seems unlikely that a response to an ML perturbation would serve only propulsion in the sagittal plane. 2) Propulsion is dependent on the stimulus direction (Fig 1C). If, for example, propulsion serves to improve stability, we would expect an increase in propulsion regardless of the stimulus direction. However, only when the perturbation is towards the trigger foot, is there an increased push-off. We see a decreased push-off (i.e. plantarflexion) when the perturbation is away from the trigger foot, suggesting a decrease in propulsion that results in an adjustment of the CoM over the base of support. Furthermore, [36] has provided evidence that ankle planterflexion torque induces trunk roll accelerations, providing further support for the push-off acting in the medial-lateral direction. Only recently has the push-off been verified to have a functional role in balance control in the medial-lateral direction as [21] show modulation of ankle torque based on CoM behavior can reduce metabolic expenditure. We speculate that the push-off is mostly acting in the anterior-posterior direction, but the offset of the feet laterally during double stance could allow for a rotation about the moment arm between the trailing limb and the CoM. Thus, the subtle modulation of push-off is less about moving the body forward and more about shifting weight to the alternate foot.

Despite the systematic nature of the push-off, the R^2^ values between push-off and other mechanisms were very small. We can provide three possible reasons for such small but consistent relationships: 1) The push-off mechanism may provide more of an indirect balance adjustment through modulation of other gait parameters, which we are currently analyzing and plan to address in a future publication; 2) The relationship between the lateral ankle and foot placement mechanisms is so strong that it masks their relationship with the push-off mechanism; 3) There are other mechanisms contributing to the maintenance of balance during walking. As discussed in the previous paragraph, we are confident the push-off is a separate balance mechanism, despite the weak correlations, in that it is activated separately from the lateral ankle and foot placement mechanisms. The small correlations between push-off and the other two balance mechanisms is actually evidence that they are separately activated balance mechanisms. For example, if the lateral ankle and push-off were activated through the same neural pathway, we would expect a strong positive relationship between the two. We actually observe a weak negative correlation between the lateral ankle and push-off mechanisms. The weak correlations may also be a product of the flexibility of the healthy young nervous system. There are so many degrees of freedom available to act on the balance related forces that a moderate amount of error at any point of the gait cycle is acceptable.

An interesting finding was that these balance responses are observed on steps both with and without perturbations. This is consistent with the view that heel strike is essentially a perturbation that is inherent to the gait cycle, requiring a control action to maintain upright balance on every step [37, 38]. This finding is substantiated by the fact that the slopes of each balance mechanism pair are not statistically different across all three stimulus directions. (*Stimulus Towards*, *Stimulus Away*, *Control*). Thus, interdependence of the balance mechanisms is present in all conditions, but the overall balance response is shifted in the direction of the perceived fall. This emphasizes that a sensory perturbation does not alter the fundamental nature of the balance response observed during unperturbed walking, but in the presence of a perceived threat to balance, the overall balance response shifts in the direction of the perceived fall (i.e. blue shifts towards the trigger foot and yellow shifts away from trigger foot in Fig 3).

The sequential onset of the balance mechanisms in the presence of the perceived fall (Fig 1) in combination with the large corrections being made for the variability of the use of earlier mechanisms (Fig 2) provides strong evidence that the CNS activates mechanisms separately based on continuous monitoring of the balance response. In this view, there would be a direct, reflex-like connection between the sensory information indicating a lateral fall and each balance mechanism, which is modulated depending on the phase of the gait cycle [39]. Since the ankle mechanism is relevant during single stance, it is possible that the effects of the ankle mechanism command could be observed via proprioception and the results fed back into the foot placement command with a negative sign. In this picture, when the ankle mechanism response is weaker due to motor noise, this would lead to stronger use of the foot placement mechanism, and vice versa, which is consistent with the correlations observed in this study. This connection of separate control loops via sensory feedback is a relatively straightforward control scheme that could explain the observed data, but it is unclear whether the proprioceptive loops could act fast enough to allow this feedback. Another hypothesis would be a multi-layered control scheme, where one layer does the high-level planning and an intermediate layer is responsible for recruiting and coordinating the low-level motor units to implement the plan. This is the view underlying the uncontrolled manifold hypothesis, that movements are planned on task level and elements on the execution-level are coordinated to implement the task-level plan by intermediate structures [40]. The uncontrolled manifold hypothesis leads to testable predictions on the structure of variance of joint angles across multiple repetitions of the same movements, where the direction of low variance is aligned with the space of joint angle changes that affects the task variable. The correlation between the ankle and foot placement mechanisms shown in Fig 2 might be a signature of such coordination, with coordination between different balance mechanisms to stabilize the CoM instead of different joint angles to stabilize the end-effector movement. Analysis of gait data using the uncontrolled manifold approach has shown that joint angles are indeed coordinated to stabilize the CoM during walking [41]. In this view, it would be possible that the intermediate level coordinates the different balance mechanisms both spatially and temporally. For instance, when the body is in a configuration due to natural gait variability where placing the swing foot more medial might bring it dangerously close to the stance leg during swing, the CNS could shift weight between the balance mechanisms to use the foot placement mechanism less and rely on the ankle mechanism more. This active coordination would be an alternative explanation for how correlation observed in Fig 2 is generated.

### Limitations

Previous work has identified different strategies that can be recruited to aid in the overall goal of maintaining stability. [42] has shown through simulations of narrow beam walking that rotations of the arms, neck, and hips can limit the angular accelerations of the head-arms-trunk complex, and thus the CoM. [43] reports that turning (i.e. foot yaw rotation) can have the same effect on medial-lateral balance as a lateral foot placement. A foot yaw response may also have an impact on the function of a push-off response, as plantarflexion through different ranges of an externally rotated foot will create different directional ground reaction forces. We did not observe such responses in the current experiment, suggesting that there may be particular situations that lead to less common but effective balance responses.

We also assume all of the modulation of the CoP with respect to the CoM in the first single stance following the trigger perturbation is a result of the lateral ankle mechanism, but it is certainly possible other mechanisms could be used to assist in this modulation. [44] also found that most of the CoP modulation is a result of ankle muscle activation, but acknowledge that the ankle responses to a perceived fall laterally is not a simple inversion/eversion activation. Future work will seek to uncover other mechanisms that can contribute to the modulation of the CoP with respect to the CoM.

We note a change in ankle plantarflexion in response to the medial-lateral balance perturbation; however, we do not have concrete evidence that the change in ankle plantarflexion angle is due to an increased ankle plantarflexion torque and propulsive ground reaction force. Due to the inherent design of our experiment, calculation of joint torques and ground reaction forces from a single limb are impossible to discern because of scenarios involving one foot on two force plates or two feet on one force plate. We asked people to treat the split-belt treadmill as a sidewalk to create an environment that allows for the most natural gait. Adding medial-lateral balance perturbations increases the number of instances where a single foot may not land squarely on a single force plate. This scenario makes it difficult to provide concrete evidence that the ankle plantarflexion response is producing a “push-off”. Future work will investigate in detail how the systematic change in ankle plantarflexion angle produces medial-lateral balance shifts, but for now we believe there is enough support to assume it does produce a CoM shift in the medial-lateral direction.

## Conclusion

In conclusion, an interdependent relationship has been uncovered between three basic mechanisms for the maintenance of upright balance during walking. Most evidently, our analysis illustrates that when the lateral ankle mechanism overshoots or undershoots the foot placement will provide a correction later in the gait cycle. The push-off mechanism is not as strongly related to the other two balance mechanisms described, suggesting the push-off is only producing a subtle balance related adjustment. Moreover, we highlight that these mechanisms are used on every step, regardless of whether there is a perturbation. The perturbations result in a shift of the overall balance response in the direction of the perceived fall. This is confirmation that a fundamental feature of human bipedal gait is a highly flexible balance system that recruits multiple mechanisms to maintain upright balance during walking despite extreme changes in body configuration and frequent perturbations.

## References

[1] Winter DA, Patla AE, Prince F, Ishac M, Gielo-perczak K, Jc Q, et al Stiffness Control of Balance in Quiet Standing. Journal of Physiology. 1998;80:1211–1221. 10.1152/jn.1998.80.3.1211 9744933

[2] Wang Z, Newell KM. Neuroscience and Biobehavioral Reviews Inter-foot coordination dynamics of quiet standing postures. Neuroscience and Biobehavioral Reviews. 2014;47:194–202. 25172292 10.1016/j.neubiorev.2014.08.007

[3] Horak FB, Nashner LM. Central Programming of Postural Movements: Adaptation to Altered Support-Surface Configurations. Journal of Neurophysiology. 1986;55(6):1369–1381. 10.1152/jn.1986.55.6.1369 3734861

[4] Peterka RJ. Sensorimotor integration in human postural control. Journal of neurophysiology. 2002;88:1097–1118. 10.1152/jn.2002.88.3.1097 12205132

[5] Oie K, Kiemel T, Jeka JJ. Multisensory function: Simultaneous re-weighting of vision and touch for the control of human posture. Cognitive Brain Research. 2002;14:164–176. 10.1016/s0926-6410(02)00071-x 12063140

[6] Robinovitch SN, Feldman F, Yang Y. Video capture of the circumstances of falls in elderly people residing in long-term care: an observational study. Lancet Author manuscript. 2013;381(9860):47–54. 10.1016/S0140-6736(12)61263-X PMC3540102 23083889

[7] Tinetti M, Speechley M, Ginter S. Risk factors for falls among elderly persons living in the community. The New England Journal of Medicine. 1988;319(26):1701–1707. 10.1056/NEJM198812293192604 3205267

[8] Berg WR, Alessio HM, Mills EM, Chen TONG. Circumstances and consequences of falls in independent community- dwelling older adults. Age and Ageing. 1997;26:261–268. 10.1093/ageing/26.4.261 9271288

[9] Patla AE, Greig M. Any way you look at it, successful obstacle negotiation needs visually guided on-line foot placement regulation during the approach phase. Neuroscience Letters. 2006;397(1-2):110–114. 10.1016/j.neulet.2005.12.016 16413969

[10] Jansen SEM, Toet A, Werkhoven PJ. Human locomotion through a multiple obstacle environment: Strategy changes as a result of visual field limitation. Experimental Brain Research. 2011;212(3):449–456. 10.1007/s00221-011-2757-1 21687987 PMC3127014

[11] Rietdyk S, Rhea CK. The effect of the visual characteristics of obstacles on risk of tripping and gait parameters during locomotion. Ophthalmic and Physiological Optics. 2011;31(3):302–310. 10.1111/j.1475-1313.2011.00837.x 21470274

[12] Donelan JM, Kram R, Kuo AD. Mechanical and metabolic determinants of the preferred step width in human walking. Proceedings of the Royal Society B: Biological Sciences. 2001;268(1480):1985–1992. 10.1098/rspb.2001.1761 11571044 PMC1088839

[13] Reimann H, Fettrow T, Thompson ED, Jeka JJ. Neural Control of Balance During Walking. Frontiers in Physiology. 2018;9:1271 10.3389/fphys.2018.01271 30271354 PMC6146212

[14] Young P, Dingwell J. Voluntarily Changing Step Length or Step Width Affects Dynamic Stability of Human Walking. 2013;70(4):646–656. 10.1016/j.gaitpost.2011.11.010 PMC3299923 22172233

[15] Yiou E, Caderby T, Delafontaine A, Fourcade P, Honeine JL. Balance control during gait initiation: State-of-the-art and research perspectives. World Journal of Orthopedics. 2017;8(11):815–828. 10.5312/wjo.v8.i11.815 29184756 PMC5696609

[16] Schrager MA, Kelly VE, Price R, Ferrucci L, Shumway-Cook A. The Effects of Age on Medio-lateral Stability during Normal and Narrow Base Walking. Gait and Posture. 2009;28(3):466–471. 10.1016/j.gaitpost.2008.02.009 PMC2583141 18400500

[17] Brach JS, Berlin JE, VanSwearingen JM, Newman AB, Studenski SA. Too much or too little step width variability is associated with a fall history in older persons who walk at or near normal gait speed. Journal of neuroengineering and rehabilitation. 2005;2:21 10.1186/1743-0003-2-21 16042812 PMC1187917

[18] Hurt CP, Rosenblatt N, Crenshaw JR, Grabiner MD. Variation in trunk kinematics influences variation in step width during treadmill walking by older and younger adults. Gait and Posture. 2010;31(4):461–464. 10.1016/j.gaitpost.2010.02.001 20185314

[19] Iles JF, Baderin R, Tanner R, Simon A. Human standing and walking: Comparison of the effects of stimulation of the vestibular system. Experimental Brain Research. 2007;178(2):151–166. 10.1007/s00221-006-0721-2 17031681

[20] Vlutters M, van Asseldonk EHF, van der Kooij H. Center of mass velocity-based predictions in balance recovery following pelvis perturbations during human walking. The Journal of Experimental Biology. 2016;219(10):1514–1523. 10.1242/jeb.129338 26994171

[21] Kim M, Collins SH. Once-per-step control of ankle-foot prosthesis push-off work reduces effort associated with balance during walking. Journal of NeuroEngineering and Rehabilitation. 2015;12:43:1–13. 10.1186/s12984-015-0027-3 25928176 PMC4429504

[22] Reimann H, Fettrow T, Jeka JJ. Strategies for the Control of Balance During Locomotion. Kinesiology Review. 2018;7:18–25. 10.1123/kr.2017-0053

[23] Reimann H, Fettrow TD, Thompson ED, Agada P, McFadyen BJ, Jeka JJ. Complementary mechanisms for upright balance during walking. PLoS ONE. 2017;12(2):1–16. 10.1371/journal.pone.0172215 PMC5325219 28234936

[24] Davis RB III, Õunpuu S, Tyburski D, Gage JR. A gait analysis data collection and reduction technique. Human Movement Science. 1991;10(5):575–587. 10.1016/0167-9457(91)90046-Z

[25] Winter D, Patla AE, Frank JS. Assessment of balance control in humans. Medical progress through technology. 1990;16(1-2):31–51. 2138696

[26] Tylkowski CM, Simon SR, Mansour JM. The Frank Stinchfield Award Paper. Internal rotation gait in spastic cerebral palsy. The Hip. 1982; p. 89–125. 7166508

[27] Bell AL, Pedersen DR, Brand RA. A Comparison of the Accuracy of Several Hip Center. Journal of Biomechanics. 1990;23(November):617–621. 10.1016/0021-9290(90)90054-7 2341423

[28] Ehrig RM, Taylor WR, Duda GN, Heller MO. A survey of formal methods for determining functional joint axes. Journal of Biomechanics. 2007;40(10):2150–2157. 10.1016/j.jbiomech.2006.10.026 17169365

[29] Lu TW, O’Connor JJ. Bone position estimation from skin marker co-ordinates using global optimisation with joint constraints. Journal of Biomechanics. 1999;32(2):129–134. 10.1016/s0021-9290(98)00158-4 10052917

[30] Dumas R, Chèze L, Verriest JP. Adjustments to McConville et al. and Young et al. body segment inertial parameters. Journal of Biomechanics. 2007;40(3):543–553. 10.1016/j.jbiomech.2006.02.013 16616757

[31] Fai AHT, Cornelius PL. Approximate F-tests of multiple degree of freedom hypotheses in generalized least squares analyses of unbalanced split-plot experiments. Journal of Statistical Computation and Simulation. 1996;54(4):363–378. 10.1080/00949659608811740

[32] Kuznetsova A. lmerTest Package: Tests in Linear Mixed Effects Models. Journal of Statistical Software. 2017;82:1–26. 10.18637/jss.v082.i13

[33] Bates D, Machler M, Bolker B, Walker S. Fitting linear mixed-effects models using lme4. Science. 2009;325(5942):883–885.19608858

[34] Hof AL, van Bockel RM, Schoppen T, Postema K. Control of lateral balance in walking. Experimental findings in normal subjects and above-knee amputees. Gait and Posture. 2007;25(2):250–258. 10.1016/j.gaitpost.2006.04.013 16740390

[35] Hof AL, Vermerris SM, Gjaltema WA. Balance responses to lateral perturbations in human treadmill walking. The Journal of experimental biology. 2010;213(Pt 15):2655–2664. 10.1242/jeb.042572 20639427

[36] Klemetti R, Steele KM, Moilanen P, Avela J, Timonen J. Contributions of individual muscles to the sagittal- and frontal-plane angular accelerations of the trunk in walking. Journal of Biomechanics. 2014;47(10):2263–2268. 10.1016/j.jbiomech.2014.04.052 24873862

[37] Bauby CE, Kuo AD. Active control of lateral balance in human walking. Journal of Biomechanics. 2000;33(11):1433–1440. 10.1016/s0021-9290(00)00101-9 10940402

[38] Wang Y, Srinivasan M. Stepping in the direction of the fall: the next foot placement can be predicted from current upper body state in steady-state walking. Biology Letters. 2014;10 10.1098/rsbl.2014.0405 PMC4190959 25252834

[39] Reimann H, Fettrow T, Grenet D, Thompson ED, Jeka JJ. Phase-Dependency of Medial-Lateral Balance Responses to Sensory Perturbations During Walking. Frontiers of Sports and Active Living. 2019;1(25). 10.3389/fspor.2019.00025 PMC7739817 33344949

[40] Scholz JP, Schöner G. The uncontrolled manifold concept: Identifying control variables for a functional task. Experimental Brain Research. 1999;126(3):289–306. 10.1007/s002210050738 10382616

[41] Papi E, Rowe PJ, Pomeroy VM. Analysis of gait within the uncontrolled manifold hypothesis: Stabilisation of the centre of mass during gait. Journal of Biomechanics. 2015;48(2):324–331. 10.1016/j.jbiomech.2014.11.024 25488137

[42] Otten E. Balancing on a narrow ridge: Biomechanics and control. Philosophical Transactions of the Royal Society B: Biological Sciences. 1999;354(1385):869–875. 10.1098/rstb.1999.0439 PMC1692595 10382221

[43] Rebula JR, Ojeda LV, Adamczyk PG, Kuo AD. The stabilizing properties of foot yaw in human walking. Journal of Biomechanics. 2017;53:1–8. 10.1016/j.jbiomech.2016.11.059 28161109 PMC6311129

[44] Hof AL, Duysens J. Responses of human ankle muscles to mediolateral balance perturbations during walking. Human Movement Science. 2018;57:69–82. 10.1016/j.humov.2017.11.009 29174418

